# THE VALIDITY AND RELIABILITY OF THE DISTRESS THERMOMETER IN FAMILY SURROGATES OF ICU PATIENTS

**DOI:** 10.1093/geroni/igac059.2045

**Published:** 2022-12-20

**Authors:** Emma O'Brien, Emily Burke, James Slaven, Tracy Taylor, Alexia Torke

**Affiliations:** Regenstrief Institute, Inc., Indianapolis, Indiana, United States; Indiana University (IU) Center for Aging Research, Regenstrief Institute, Inc., Indianapolis, Indiana, United States; Indiana University, Indianapolis, Indiana, United States; Regenstrief Institute, Inc., North Vernon, Indiana, United States; Indiana University (IU) Center for Aging Research, Regenstrief Institute, Inc., Indianapolis, Indiana, United States

## Abstract

Brief, reliable assessment tools are highly valued in both research and clinical settings. The single-item Distress Thermometer (DT) asks participants to rank their overall level of distress from zero to ten. Similar measures of distress perform well in oncology populations, but the validity of the DT has not been well tested with other populations. To determine its validity and reliability, we analyzed data from family surrogates (n=188) of critically ill ICU patients. Surrogates were asked to rate their distress during the first four days of the patient’s ICU stay and 6-8 weeks after discharge (n=127). Data were analyzed using Spearman non-parametric correlation due to the distributions of the data. DT scores at both baseline and follow-up were significantly correlated with anxiety (GAD-7: correlation coefficient (ρ)=.527, p<.0001; ρ=.543, p<.0001, respectively), depression (PHQ-9: ρ=.480, p<.0001; ρ=.399, p=.0002), distress (Kessler-6: ρ=.477, p<.0001; ρ=.528, p<.0001), and negative religious coping (ρ=.149, p=.0426; ρ=.238, p=.0074). Results also indicated that spiritual well-being at baseline and follow-up (FACIT: ρ=-.391, p<.0001, ρ=-.443, p<.0001) and positive religious coping at baseline (RCOPE: ρ=-.164, p=.0253) have an inverse relationship with overall distress. At baseline, surrogates with better positive religious coping and/or more involvement in organizational religious activity (ρ=-.189, p=.0106) were more likely to report lower distress. The DT could be an efficient, single item predictor of outcomes that impact patient and family care. Future research could confirm its validity as a measure of distress, in a variety of clinical populations and environments that could inform clinical care for patients and families.

